# Transition Needs Compassion: a Thematic Analysis of an Online Compassion-Focused Therapy Group in a Gender Service

**DOI:** 10.1007/s12671-022-01893-9

**Published:** 2022-05-04

**Authors:** Alastair Pipkin, Aimee Smith, Christina Shearn

**Affiliations:** 1 Northamptonshire Gender Service - Northamptonshire Healthcare NHS Foundation Trust, Danetre Hospital, London Road, Daventry, NN11 4DY UK; 2 Northamptonshire Healthcare NHS Foundation Trust, Daventry, UK

**Keywords:** Compassion-focused therapy, Gender diversity, Group therapy, Transgender and gender non-conforming, Stigma, Self-acceptance

## Abstract

**Objectives:**

Transgender and gender non-conforming people may face elevated rates of shame and self-criticism in light of minority stress. Compassion-focused therapy has a growing evidence base in addressing trans-diagnostic processes in mental health difficulties, including shame and self-criticism. The objective of the present study was to explore the experience of an initial pilot compassion-focused therapy group delivered online in a Gender Service during the COVID-19 pandemic.

**Methods:**

Six transgender people completed a semi-structured interview after attending an 8-week compassion-focused therapy group in a national Gender Service. Inductive thematic analysis was used to identify themes in the data.

**Results:**

Four themes were identified from the data: *Transition Needs Compassion; Acceptability of the Compassion-Focused Approach; Being in a group with other transgender people; and Online delivery works despite its challenges.* Participants reported that the compassion-focused framework was an appropriate and helpful way of understanding their experiences of stigma and that both the content and process of the group had benefitted them. Being with other transgender people raised some anxieties, such as comparisons or fear of offending, but also enabled seeing the self in more positive and accepting ways. While online delivery had some challenges, participants largely felt it was an effective mode of delivery, aided by the experiential nature of the group.

**Conclusions:**

Compassion-focused therapy seems to be a feasible and acceptable approach for transgender and gender non-conforming people. Group processes may be helpful in increasing self-acceptance. Further quantitative exploration of therapy process and outcomes is warranted.

Transgender and gender non-conforming people (TGNC) experience elevated rates of stigma and discrimination due to holding a minority identity, which is a known precursor to psychological distress (Scandurra et al., 2019). Minority stress theory (Meyer & Frost, 2013) proposes that holding a minority or stigmatised identity can become internalised to increase the risk for psychological distress, known as internalised stigma (Hendricks & Testa, 2012). Internalised stigma may constitute shame for oneself or aspects of identity (Hatzenbuehler & Pachankis, 2016). A wide range of research reports increased rates of psychological distress for those with minority identities (Dhejne et al., 2016), and self-criticism and shame may be some consequences of stigma (Hatzenbuehler & Pachankis, 2016; Vincent & Lorimer, 2018), both of which are known trans-diagnostic factors in mental health difficulties. It is therefore important to better understand how to address the psychological mechanisms contributing to internalised stigma in groups at elevated risk to promote psychological well-being.

Self-compassion is defined as the capacity to be open to, moved by and motivated to alleviate one’s suffering (Neff, 2003). Research has operationalised self-compassion using three components (Neff, 2003; Neff & Tirch, 2013). Self-kindness versus self-judgement, or the extent to which one holds unconditional positive acceptance for oneself in the face of suffering, pain, failures or inadequacies versus an over-engagement with self-critical or harsh self-judgements. Common humanity versus isolation, referring to the extent to which one views the human experience—including suffering, failures and inadequacies—as inclusive and broadly shared or universal, rather than experiencing the self as somehow different or alone. Mindfulness versus over-identification, referencing the extent to which one can recognise thoughts and feelings from a curious, non-judgemental stance without reacting to them. Such processes are proposed therapeutic mechanisms in compassion-focused therapy (CFT; Gilbert, 2009), which is a third-wave behavioural therapy focused on fostering the above skills through psychoeducation, mindfulness skills and a range of experiential exercises to develop self-kindness and a sense of common humanity. CFT is commonly delivered in a group format, such as Compassionate Mind Training (Gilbert & Procter, 2006), which involves modules covering the above elements, alongside group-based exercises to promote the interpersonal experiences of common humanity, mindfulness and kindness. CFT has a growing evidence base as a psychological treatment for shame, self-criticism and limited skills in self-compassion (Cuppage et al., 2017; Gilbert, 2009), although this study is limited by short-term follow-up measurements of 2 months. Recent meta-analytic research demonstrates that self-compassion-focused therapies, including CFT, are effective at improving self-compassion and psychopathology (Wilson et al., 2019), and at improving mindfulness skills and reducing depression and anxiety (Kirby et al., 2017). Broader studies examining the processes underlying CFT suggests that self-compassion moderates the impact of psychological processes commonly found in mental health difficulties, such as moderating the association between rumination and stress (Samaie & Farahani, 2011). Research therefore suggests that developing the skills for self-compassion influences a range of psychological and emotional processes which otherwise contribute to distress.

Some research has begun to explore the role of self-compassion in minority stress, with recent research proposing that self-compassion may mediate and moderate the impact of stigma (Wong et al., 2019). Using Neff’s (2003) three components, internalised stigma may operate by contributing to excessive self-judgements rooted in experiences of discrimination, over-identification with stigma-based narratives and a limited sense of common humanity due to a perceived sense of inadequacy or isolation (Wong et al., 2019). Self-compassion has been shown to moderate the relationship between gender non-conformity and subjective well-being in a community sample (Keng & Liew, 2017). It has further been shown to co-vary with marginalised identity status and therefore may be a protective factor against the impact of discrimination and stigma (Vigna et al., 2018). To this end, targeting self-compassion in psychological therapy with TGNC people may function as an appropriate trans-diagnostic intervention for the range of psychological difficulties they may experience, including negative self-concept and self-criticism (Austin & Craig, 2015; Scandurra et al., 2019), and may also bolster resilience towards minority stress and mental health difficulties through fostering mindfulness skills, self-kindness and common humanity. CFT may therefore be an appropriate intervention to explore further in this field.

Within the UK context, TGNC people may access one of seven national Gender Services, which provide medical gender-affirming interventions such as hormone therapy and psychological therapy to address any gender-related psychological concerns (NHS England, 2019). Psychological therapy groups promoting psychological well-being are routinely delivered, such as groups aimed at improving confidence, social transitions or emotional well-being. CFT was deemed an appropriate intervention as part of routine practice to address self-criticism and shame as contributory factors to psychological distress, with adaptations made to consider the specific needs of TGNC people. In this context, the approach considered cultural-specific issues such as considering early experiences of gender dysphoria, coming out and trans-discrimination as possible shame memories, and considering how stigma and minority stress may impede positive self-to-self and self-to-other relating (Gilbert, 2009). The CFT approach considered Wong et al. (2019) proposed model of self-compassion to address internalised stigma, which suggests that stigma becomes internalised through increasing self-criticism, negative affective responses and reducing social support. The model proposes that fostering positive self-judgements versus over-identifying with internalised stigma or self-critical narratives, developing emotional regulation skills and fostering social connections therefore reduce the impact of stigma.

The present study presents a qualitative evaluation of a pilot CFT group run online in a Gender Service in England. It aimed to explore the experience of attending the 8-week, structured CFT group with TGNC people. The study explored the following questions: (1) how do people accessing a Gender Service experience a CFT group intervention? and (2) what is the experience of a CFT group intervention run online?

## Method

### Participants

A total of ten participants completed the group, of which six participants took part in the present service evaluation study. Reasons for not taking part included being discharged from service and unable to contact (*n* = 1); and not being able to take part due to competing life demands (*n* = 3), one of which was due to accessing other services following completion of the group and two of which were due to on-going social issues during the conduct of the study. All participants were TGNC people attending a Gender Service in England. The age range was 30–54, they were all white British, three identified as transgender women, two as transgender men and one as non-binary trans masculine. They were all accessing medical and psychosocial support at the Gender Service. All had been identified as potentially suitable for a group intervention and had therefore been placed on the group waiting list. While diagnostic interviews were not undertaken, at initial assessment a semi-structured clinical interview and use of routine psychometric outcome measures determined they were presenting with a range of concerns, including mild depression (*n* = 3), social anxiety (*n* = 2) and generalized anxiety (*n* = 1) and low self-confidence (*n* = 4).

### Procedures

The group waiting list was contacted in full to ascertain those interested in attending a group. Fifteen people were agreeable to talk further, of which five declined, with primary reasons being too anxious at this time (*n* = 2); too busy/unable to attend regularly (*n* = 3). Initial assessments were conducted individually to identify goals; main presenting concerns; any risk issues or wider concerns; and to prepare for the group and identify any worries or issues, such as with the use of Microsoft Teams. Participants were recruited to the group on the following inclusion criteria: presenting needs regarding their emotional well-being; willingness; and ability to engage in the group intervention. The only explicit exclusion criteria were the presence of acute risk concerns which would require more specialist mental health support and an inability to attend all eight planned sessions. Following the initial assessment, nobody was identified as inappropriate for the group context. Subsequent inclusion criteria in the present study was willingness and ability to talk about their experiences; there were no explicit exclusion criteria.

The eight-session group was delivered on a weekly basis for 2 h via Microsoft Teams, with a 10-min break in the middle. All three authors were co-facilitators. None of the facilitators were TGNC themselves, though one belonged to the LGBTQ + community, one was male, two were female and all three were White British. All participants received the same intervention and home practice activities. Post-group follow-up sessions were conducted individually when the post-group outcome measures were completed.

The group protocol was developed by the lead author and was based around Compassionate Mind Training group protocol (Gilbert & Procter, 2006) and a similar pilot with sexual minority people (Pepping et al., 2017), which adapted Compassionate Mind Training using sexual minority-specific issues and discussion points (see Table 1 for session content). The group protocol contained a mix of experiential, didactic and discursive exercises and weekly home practice tasks from four modules: psychoeducation about compassion (e.g. evolutionary theories, ‘tricky brain’, qualities of compassion, barriers to compassion, three-circle model); body-focused interventions (e.g. soothing rhythm breathing, body posture, tone of voice); mindfulness; and developing the compassionate self (e.g. imagery exercises, safe place, flows of compassion exercise). TGNC-specific adaptations included discussion around the impact of stigma, TGNC-specific life experiences or such as coming out or early experiences of gender dysphoria and discussions of the impact of societal narratives and learned safety strategies.

**Table 1:** Details of the session content and home practice activities

Session	Session content	Home practice
1	Introductions, group rules Qualities of compassion The three flows of compassion ‘Tricky brain’ and the old brain/new brain concept Mindfulness	Noticing the three flows of compassion Mindfulness practice
2	Barriers to compassion exercise Exploring internal dialogue and self-criticism Exploring trans-discrimination and stigma as contributors Soothing rhythm breathing	Beliefs about self-compassion and self-criticism exercise Practice soothing rhythm breathing
3	The three-circle model Identifying threats—internal and external Exploring the function of self-criticism	Reflection on own three circles Functions of self-criticism exercise
4	Exploring safety strategies and unintended consequences Group flows of compassion exercise Safe place imagery	Practice safe place imagery Identifying own safety strategies
5	Trans-discrimination and internalised transphobia Compassion-focused formulation exercise using TGNC example Grounding exercises	Fill in own formulation Practice grounding exercises
6	Developing a compassionate internal dialogue Facing and managing emotions Managing internalised stigma narratives Compassionate Companion imagery exercise	Practice Compassionate Companion imagery Create self-soothe box
7	Qualities of self-compassionate internal dialogue Compassionate letter writing	Compassionate letter writing to self Write compassionate comments for each group member
8	Reflections on group Developing ‘keeping compassion going’ plans Group flows of compassion exercise	Fill in ‘keeping compassion going’ plan

A semi-structured interview schedule was co-created with Experts by Experience within the service, who were five TGNC people accessing the service who meet monthly to inform service development. This was utilized to expand user involvement in the study and consider the relevance and suitability of the questions posed (see Table 2 for interview questions). Microsoft Teams was utilized to deliver the group and conduct the interviews.

**Table 2:** Interview topic guides and suggested prompts

Topic guides	Prompt areas
What made you join the group?	
What was it like being in the Compassion Group?	Content? Set-up? Pace? Improvements?
Was anything about the group helpful? If so, what was it?	Did anything change for you? What do you think about the concept of self-compassion in the Gender Service?
Has anything stayed with you since you the group?	More/less flows of compassion?
Was anything challenging about the group? If so, what was it?	Content? Set-up? Pace? Improvements?
Is there anything else you would like to say about the group?	Expectations?

Following ethical approval, all group attendees were emailed the details of the study, which occurred 3 months after the completion of the group. The six participants who took part were provided with the opportunity to provide informed consent, and an online interview appointment was arranged at a suitable time. The interview schedule (see Table 2) explored how the experience of the group and its content had been, how attending online had been and general feedback, though largely followed participants’ leads. It was utilized in a semi-structured fashion by introducing broad topic areas, but largely following participants’ leads using prompt questions for further information. Interviews were conducted via Microsoft Teams by the third author, and they were recorded with participants’ permission and informed consent. Recordings were deleted following verbatim transcription. The third author who conducted the interviews was a facilitator for the group sessions; potential bias here is acknowledged in light of service constraints, though active efforts were made to ensure constructive and open feedback was given, such as prompting for challenges and difficulties.

### Data Analyses

The study used a qualitative, cross-sectional design with a self-selecting sample and used inductive thematic analysis (Braun & Clarke, 2006). The researchers held a critical realist epistemological position (Fletcher, 2017), acknowledging that researcher reflexivity informs the consideration and interpretation of the data, but that a reality exists ‘out there’ which may be observed.

Braun and Clarke’s (2006) inductive thematic analysis framework was considered apt to ascertain themes in the participants’ experiences of the group. It followed the six phases outlined by Braun and Clarke (2006). Further analytical guidance was considered from Nowell et al. (2017).

Once interviews had been completed, the third author transcribed all transcripts verbatim. Initial bracketing exercises were completed by the research team using written prompts, during which all three authors noted their critical realist epistemological assumptions and theoretical interests in models of internalised stigma. Attempts were made through each subsequent phase and the team supervisory meetings to bracket such assumptions from the reading of the data. The first and third authors further kept reflective diaries during the analysis phase to support researcher reflexivity and the bracketing of such assumptions.

Phase 1 involved the first and third authors reading both transcripts twice in full and noting initial points of personal or professional resonance, which were documented separately as part of reflexive notes to enable consideration of potential biases in interpretation. These were discussed and compared during initial team meetings. Phase 2 involved identifying each unit of meaning and attaching a short verbal code name to reflect what the participant was referring to, e.g. ‘Online delivery was challenging’. Authors 1 and 3 individually coded each transcript and subsequently met to discuss and compare codes, during which biases, discrepancies and closeness to the original data were discussed as codes were refined. Minutes of meetings were kept alongside bracketed reflexive notes from phase 1 to enable an audit trail. The second author further reviewed each set of codes for each transcript to support researcher triangulation. A further team meeting enabled peer debriefing and consideration of credibility, after which each set of codes for each individual transcript was agreed upon.

Codes were then listed and organized into initial themes per transcript for phase 3, with codes referring to similar concepts being grouped into themes. This was a reiterative process to best capture the meanings expressed by each participant, and final thematic structures were agreed upon by all three authors. Phase 4 extended the reiterative process of theming to identify an overarching thematic structure incorporating codes from all transcripts. During this phase, the team re-organized codes into a proposed final thematic structure. A small number of codes within existing themes were removed as they were deemed to not have relevance, e.g. codes pertaining to experiences of gender medical care in England. No additional codes were added upon reviewing the audit trails and overall data set.

Phase 5 involved naming and defining the themes, which were agreed by all three authors and checked with participants. Theme trustworthiness was considered through credibility checking, though an acknowledged limitation is the lack of further consideration of data saturation, partly due to the limited sample size. The team agreed that similar content was identified across the interviews and that no new themes were identified in the latter interviews, though further exploration is needed with larger sample sizes. Narrative summaries of each theme were written with direct links to the original data and code structure. The thematic structure with illustrative quotes is outlined in the “Results” section.

## Results

The thematic analysis identified four super-ordinate themes: (1) Transition Needs Compassion; (2) Acceptability of the Compassion-Focused Approach for TGNC issues; (3) Being in a group with other transgender people challenges assumptions; and (4) Online delivery worked despite the challenges. The second theme contains two sub-themes: (2.1) Experiences of the content and (2.2) Experience of the process. Each will be outlined in turn using illustrative quotes agreed upon by all three authors. Quotes are presented verbatim with additional context added for clarity using [] where required.

### Transition Needs Compassion

All six participants talked in broad terms about the emotional impact of their gender transitions and the need for psychological support to address this. Participants spoke of a range of emotional challenges during their transition, including finding it difficult to *“fit in”*, challenges with finding their *“identity”* among stigma and social exclusion and *“traumatic experiences”* to do with delays and disruptions to their medical care. Four participants spoke explicitly about this line of support being neglected in Gender Services, where they had experienced medical transition take primacy over exploration of their emotional well-being:

*I was always very worried when I left [Gender Service], I had 5 years with no contact with anyone in terms of emotional support, no physical support, and it’s like “well you’ve had everything done now, you don’t’ want the other operation so you may as well get on with your life then”. And you think, ‘oh great, I’m fine’, but then a year in you start to feel wobbly, and you struggle with things, because whether you like it or not you’ve gone through a totally different experience than everyone else around you. Whether you identify as male, female, non-binary whatever, you’ve gone through a totally different experience in terms of gender recognition to everyone else around you, and that impacts every decision in your life in terms and what you go through, and to not have the emotional support there from a clinician or a group I think is really dangerous, and neglectful as well.* Oliver

Four participants further expressed a sense that internalised transphobia resulted in a tendency to focus *“externally”*, such as to compare oneself to others, to hold concerns about ‘passing’ as a particular gender identity and to be preoccupied with worries about their transition journey and the risk of experiencing discrimination or social isolation. Three participants commented that the CFT model’s encouragement to focus internally and to address self-criticism and negative self-beliefs and the space to process difficult emotions related to their transition journey was a welcome line of support that had otherwise been missing in their experience thus far. One participant stated that *“compassion is the antidote; the opposite of internalized transphobia”*, that it enabled *“addressing the hurt of stigma”* by processing emotions and considering one’s internal dialogue:

*We’re taught how not to do it, you know* Jake

Two participants commented that self-compassion felt important in addressing their concerns with ‘passing’ and addressing the *“self-resentment”* felt from a gap between their *“inner and outer self”*.

Overall, all six participants noted that the concept of self-compassion felt missing throughout their transition journeys, with its attention to internal experiences, its focus on self-affirmation and emotion regulation. Social isolation was a concern for all of the participants during their journeys.

### Acceptability of the Compassion-Focused Approach for TGNC Issues

#### Experiences of the Content

All participants reported finding the CFT model an accessible way of understanding their emotional worlds and the impact of some of the challenges pertaining to their transition. Most participants noted that exploring the barriers to compassion, understanding how the self-critic functions and considering the three-circle model were helpful in raising their self-awareness of these issues in their day-to-day life. They acknowledged that some of the skills-based content had stuck with them more than others, which for most participants translated to two or three techniques and concepts which they still used at the time of the interview. For example, some participants reported the Compassionate Imagery exercises to be particularly helpful, reporting that the exercises enabled them to access positive, affirmative self-statements and soothing feelings:

*Compassionate companion was immense, I have issues with self-esteem, I expect lots of trans folk do, self-compassion is something I’ve struggled with. Life has been difficult outside all of this and it continues to be, so I’ve been using the tools to remind myself that I’m doing okay and that how I’m organising my time is okay. It’s been incredibly useful.* Ryan

*You know there’s one of them that I still use now, it’s the umm, compassionate friend, is it? You know that talks to you and just gives you a bit of… gentle encouragement, and a bit of… stability, you know, makes you feel better. I go ‘uhhh thank you for saying that!’. I know it’s myself but it’s strange how you go to – for me it was my dog.* Sandra

For other participants, the Compassionate Comments exercise was powerful:

*The lovely compassionate comments from everyone – blew my mind. I just cried. I was really moved because- I’m sure everybody felt that but especially maybe with the internet version of it, that it’s difficult to get a sense how you’re landing, and you could see that how many people apologised after speaking, so to get that back – sort of warmth and appreciation - was genuinely a surprise. I definitely don’t see myself the way other people see me, so that was very powerful and I’m sure it was the same for everyone.* Jake

Two participants felt that the Loving Kindness meditation was a *“stand-out”* moment, relating it to a direct experience of feeling compassion from the group as well as an opportunity to face the barriers as they occurred. Four participants described benefitting from learning about and being more able to directly challenge self-criticism:

*There are times when I – you know, something comes up from the past – I mean I’m full of bad stuff from the past and ‘oh you’re an idiot’, you know, and like ‘yeah, well, actually no you’re not, that was 40 years ago’, you know. It told me to concentrate on those things, you know. I mean, that could… spiral, in the past.* Lucy

Participants all felt that the content and skills taught enabled learning about how to engage with self-compassion, and they all talked about addressing some of the barriers to accepting self-compassion following the group.

Overall, the participants described the concepts to be accessible and practically useful, with all participants noting a couple of strategies which had a tangible impact on their day-to-day life. The majority added that the CFT model felt relevant for trans-specific issues, such as low self-esteem, shame and self-criticism and difficulties connecting with others due to fear of judgement.

#### Experiences of the Group Processes

All six participants described helpful and positive experiences with the CFT group processes. Five participants commented on a lasting sense that *“we’re all in this together”* following various group discussions about shared experiences and the Loving Kindness meditation. For most participants, this came from feeling *“grounded”* or *“okay”* in oneself through connecting with others, which they felt helped their self-acceptance:

*I learned that erm, there isn’t one kind of trans girl or trans guy, you know, it is a massively varied kind of, well they talk about the gender spectrum obviously, and actually that has really helped me, so that was a really good by-product because actually I can just be who I want to be and that doesn’t have to be a PVC dress wearing wig make-up trans girl, which some people are absolutely fine in doing, I can wear jeans a hoodie and converse and still be happy about who I am.* Jamie

The process of being with other transgender people appeared to complement the content and went some way to challenge negative stereotypes or expectations they felt they needed to live up to. Their descriptions seemed to relate to developing a sense of common humanity from recognizing and connecting with diversity in the TGNC community. For Ryan, the sense of bonding and compassion within the group, aided by the Loving Kindness meditation and bonds which developed outside of the group, left him with a sense of being worthwhile and cared for:

*To have that feeling of connection and support even if it’s invisible, you know I can feel that it’s there. I can either escalate that to the point I’m panicking about it or I can think, ‘ok this is a situation I’ve got to fix, I’ve got people who care about me, people I’ve just met who actually care about me enough to join this Whatsapp group..’. it’s like, ‘ok there are good people in my life who don’t think I’m worthless or crazy’.* Ryan

Three participants commented on the use of validation of feelings, self-disclosure and humour by the facilitators:

*When [Facilitators] were sort of participating very openly, you know, being quite vulnerable and you know, role-modelling that and I know that’s always very powerful when you see that, it gives permission, it shows you that it’s possible, but I think you took the course deeper really quickly. My sense is there was a ripple of (sighs deeply) ‘okay’, you know. So that helped me, it helped me but I think it helped all of us.* Jake

It is notable that the majority of the participants referred to a *“depth”* in the group despite it being online; facilitator techniques such as self-disclosure and modelling and experiential emotion-focused exercises seemed to contribute to this from participants’ accounts. Participants described that the continued modelling of mindful noticing and validation of feelings helped to address their tendency towards negative self-judgements.

Overall, participants’ experiences reflect that various CFT therapeutic processes aided a sense of safety and went some way to challenge their negative beliefs about themselves. The experiential focus seemed to aid this and participants’ found the CFT model a helpful way to consider their experiences of being transgender, such as understanding the impact of stigma and discrimination on their threat responses and self-criticism. Group-based experiential exercises seemed to foster a sense of common humanity in line with the CFT model, which participants felt had relevance for developing their self-compassion as the group progressed.

### Experience of being in a group with other trans people

All participants commented on the benefits of feeling connected with a group of TGNC people. This was termed as a *“support network”,* an *“authentic space”* and an experience of *“bonding”.* This was not without its challenges, with five participants expressing persistent worries about how they were *“coming across”* and whether they would *“offend”* or *“do something wrong”*. For Jamie, attending a group at the Gender Clinic raised particular anxieties about their transition journey:

*The fact that I hadn’t really started my [transition] journey, I struggled with that because I met with people, the only other people who hadn’t [transitioned] were [facilitators]. So that made me think, ‘oh Christ what are they gonna think about me’, because I’m just this massive hairy bloke’ish sounding bloke called [name] in a trans group, and that kind of made me feel a bit anxious.* Jamie

While fear of judgement and negative comparisons to others may be common groups processes, four participants commented on transgender-specific anxieties, such as who was earlier or later in the journey and how they may be viewed. Oliver experienced challenges from facing memories of his own transition journey through hearing other peoples’ stories and struggles:

*The second thing is how I’d come across in the group and not knowing what stage everyone else is at, cos that was a bit of a concern, and there were times within the group where I felt that was good in some ways because it helped the people coming through the system earlier on feel better, but for someone higher up who’s been through it was harder to go back over those memories and go through it again.* Oliver

This seemed to relate to a shared concern among participants about what others will think or feel in response to their place in the world, be it their transition ‘stage’, their gender expression, or what opinions they held. Participants referred to self-monitoring and feeling distracted by their preoccupation with what other people will think or feel in reaction to them, their opinions or their experiences.

Some participants felt that the group raised various sub-group dynamics within the trans community and others queried whether separate gender groups may be helpful or more divisive.

*It’s interesting having trans men, trans women, trans masculine and non-binary all in the same group. And it might be good to find a way, or perhaps it’s not perhaps it isn’t good to highlight those divisions, but there are differences and there does need to be appreciation I think probably from the facilitators perhaps more than the participants of those particular delineations. In the community there’s a trope than trans men and trans masc people don’t speak up, and someone said something along those lines and yet they were dominating the conversation, and I think that would be good to be aware of in future groups. You know I’ve been raised not to speak up, not to have a voice, other people have been raised to be the centre of attention and to be listened to, and that doesn’t mean I’m hiding in my male privilege and being sexist or that trans women are doing all the work, it just means that I would appreciate those deep experiences I have having the same floor as the louder folks, and they can be from any gender or genders.* Ryan

They highlighted the importance of facilitators’ attending to historical and indeed current experiences of discrimination and how these dynamics may enact in the therapy group. Overall, being with other transgender people raised opportunities for connection, developing self-acceptance and feeling part of the community. It also raised various levels of anxiety, including fearing negative evaluation, facing up to one’s own identity, re-experiencing dynamics of discrimination or sub-group dynamics and comparing oneself to others.

### Online delivery worked despite its challenges

The final theme relates to participants’ experiences of the CFT group being run online. All participants reported an overall positive experience of the group being run online, though four described initial anxieties adjusting to the format. Some felt it made for a more comfortable atmosphere in ways:

*I think in a way it made people more open, which is really strange, I found that people were more comfortable about having an open conversation about things, I don’t know why, maybe because you’re in your own space, I’d be very interested to see how differently we’d all have reacted being in a big circle in the same room. So I think, yeah it had pros and cons, definitely setting it up and that was hard, but once we got used to it, it was fine for me, I didn’t find it uncomfortable. The only time I had to turn my camera off was the meditations, that felt a little bit strange having my eyes closed with people watching me, but the rest of it was fine, no issues at all.* Oliver

However, some participants had a differing experience and commented on concerns for privacy and how this became distracting within sessions and disrupted engagement:

*I was a bit wary about what I wanted to say. It was very, umm, some of it lost me a little bit, but I think that was due to being sort of wary of my environment on this side.* Sandra

Participants became aware of the need to ensure a private, comfortable space was available to take part. Jake also found the intrusion into other peoples’ homes, particularly their bedrooms, “*intrusive”* and “*uncomfortable”* at times.

Three participants commented on additional anxieties relating to the online functionality of the group. Being able to access the breakout rooms, worrying about talking over people or somehow messing up with the technology was a fear throughout the group for most participants:

*I never quite figured the interface for just bouncing between rooms and uhh… I did find that very very stressful and I also found the not quite knowing how much time we had a bit stressful, but I think that’s a personal stress thing is that, you know, there are times when things will just stress me, you know.* Lucy

For two participants, dysphoria was a concern, particularly through being able to see themselves on screen for the entire group, which they noted may be less of an issue face to face. Sandra found this particularly distracting until she figured she could cover that part of her screen, which helped. Lastly, two participants noted challenges with being on a phone or an old computer:

*“I couldn’t read how the whole thing was going down which in a group I could and adjust accordingly, so that was difficult. That was my Microsoft Teams set-up with an old computer and everybody else could”* Jake

This provided some disruptions to their ability to see and engage with everyone. The group happened around the time Microsoft Teams had increased viewing capacity to nine people at a time for most computers, though some struggled with not having this capacity.

In summary, while the participants largely reported a positive overall experience of the group in terms of both content and process, and positive self-reported outcomes were evident for all, online functionality issues, confidentiality and dysphoria may pose additional factors for negotiation in the delivery of online therapeutic groups.

## Discussion

The thematic analysis reported four themes, overall detailing that an online CFT group was experienced as acceptable and appropriate by the participants. All participants referred to the need for psychosocial support during and after gender transition, as negative self-concept, social isolation and emotional difficulties were commonplace in their experience but often not addressed. Anxieties were noted about the online delivery, such as being able to see oneself, having less non-verbal feedback from others and worrying about the tech. Participants also shared anxieties about being in a group with other TGNC people, including comparing oneself to others, facing up to one’s identity and fearing offending others. The group processes—namely use of experiential exercises and a sense of bonding—appeared to alleviate some of these anxieties and, to participants’ minds, address some of their negative beliefs.

### Compassion-Focused Therapy for Gender-Related Issues

Participants all felt that CFT was an appropriate and relevant approach for issues such as low self-esteem, which the majority linked to experiences of stigma and discrimination. The findings seemed to speak to the direct application of the CFT model to internalised stigma, such as participants entering the group with anxieties about perceived differences and anticipated judgements falling away to a sense of common humanity, and increasing mindfulness skills and self-kindness enabling de-identification with negative stigma-based beliefs. This echoes the growing field of research suggesting that self-compassion may be a suitable target for intervention in TGNC people and may go some way to address the impact of stigma (Wong et al., 2019). Participants also felt that the model helped them to understand broader issues such as low mood, self-confidence and anxiety, suggesting it may be an appropriate trans-diagnostic intervention as in other fields (Cuppage et al., 2017). While therapy process and outcome research remain limited, further research can expand on prior studies to examine whether self-compassion indeed buffers the impact of stigma and discrimination (Vigna et al., 2018) and whether it is therefore a viable psychological treatment target for TGNC people in addressing negative self-concept (Austin & Craig, 2015). Processing issues such as self-criticism and stigma with other TGNC people may contribute positively to the therapeutic processes, such as challenging stereotypes and negative beliefs about oneself in the context of validation and acceptance. In light of broader research suggesting that social connection has a positive impact on the well-being of TGNC people (Bowling et al., 2020; Budge et al., 2014), therapeutic approaches and spaces can incorporate the potential benefits of peer learning and group processes.

More broadly, participants all referred to the emotional impact of their medical gender transition. While progress has been made with regards to both the understanding of the emotional impact and provision of support (e.g. all Gender Services in England are commissioned to provide direct psychosocial support alongside medical interventions; NHS England, 2019), there is a broader point for clinicians supporting TGNC people to enquire about the emotional impact of transition. Participants here referred to a range of experiences, such as feeling different from those around them and social isolation (i.e. lacking a sense of common humanity), struggling with over-focusing on the external world and neglecting their own emotional experiences (i.e. over-identification rather than mindfulness) and challenges related to direct experiences of discrimination and navigating the medical system (i.e. less external experiences of kindness allowing for less internalised self-kindness). Participants referred to a range of CFT exercises raising their awareness of and ability to address some of these issues, specifically group-based self-kindness exercises such as Loving Kindness and Compassionate Comments, and mindfulness skills to attend to their experiences. A CFT framework may be helpful in formulating the extent to which transphobia and difficult experiences with coming out and beyond result in less self-compassion, though at this stage this requires further empirical investigation regarding its utility.

### Trans-Specific Therapy Groups

The present findings also raise considerations for TGNC-specific groups. The majority of participants highlighted anxieties around comparison and the fear of offending others, and some were specifically reminded of particular stereotypes or dynamics from prior experiences. Prior research into transgender-specific therapy groups suggests that facilitators ought to attend to the risk of subgrouping, and that considering how wider cis-normative assumptions may result in a pressure to conform or present oneself in a particular way within the therapy room (Heck et al., 2015). The findings here contribute to these considerations, therapists may need additional cultural sensitivity to the different layers to the TGNC experience and how this may enact in the therapeutic space. For example, non-TGNC facilitators may need an additional awareness of their own position to cis-normative assumptions, and to be aware of the risk of gender-based norms and assumptions feeling enforced in the minds of the participants. The present findings suggest that TGNC-specific therapy groups may provide some space for addressing this, though this warrants further investigation. Engaging with the TGNC community may help to actively attend to and address such issues of how difference emerges in therapy; some suggestions include seeking input from user involvement initiatives regarding how to facilitate safe spaces, co-facilitation by a member of the TGNC community and exploring issues of subgrouping and conformity with the group itself, acknowledging where cisgender facilitators are in place. The findings here suggest that these may be important features of TGNC-specific therapy groups and can be used for therapeutic benefit by challenging stereotypes, assumptions and stigma-based beliefs. This warrants further investigation and attention in clinical practice.

### Online Delivery

The findings suggest that online delivery of a CFT group felt acceptable and that all participants reported taking some experiential benefit from the process of the group. A recent review of the research on online therapy groups suggests that therapists can increase the use of self-disclosure and the use of experiential strategies to support engagement and outcomes, though research remains scarce (Weinberg, 2020). The findings here support this; CFT in particular is a process-focused approach with ample use of experiential, discursive and group-based activities. Microsoft Teams, although bringing some anxieties, appeared to enable therapeutic processes to occur from the participants’ accounts. Further quantitative evaluation of the clinical outcomes is needed.

### Limitations and Future Research

The present study is limited to its small and self-selected sample; four of the original attendees did not opt to take part in the study which may have limited diverse experiences being heard. The study also utilized semi-structured interviews which is a limitation, due to the scope for influence on interviewees’ responses through the use of follow-up, leading or suggestive questioning. Data saturation was not considered in depth due to it being an initial evaluation and the limitations of a small sample. While similar themes were identified across the six interviews, this is an acknowledged limitation, and larger studies across different settings are needed. Being qualitative in nature, the data are limited regarding generalizability and does not account for clinical changes from the group beyond reporting subjective experiences. Power imbalance is also acknowledged, as resource limitations meant that the third facilitator conducted the interviews, which reflexively may have restricted some details being shared. Wider user involvement was also limited due to service constraints, participants or Experts by Experience co-coding and analyzing data would have further supported its validity.

## Data Availability

Due to being qualitative in nature, data are not available to protect the anonymity of the participants.
